# Sexual behavior of women who have sex with women

**DOI:** 10.61622/rbgo/2026rbgo34

**Published:** 2026-07-17

**Authors:** Debora Aiesha Leite Cantelli, Sérgio Henrique Pires Okano, Luiz Gustavo Oliveira Brito, Rosana Maria Reis, Ana Carolina de Sá Rosa-e-Silva, Lúcia Alves da Silva Lara

**Affiliations:** 1 Universidade de São Paulo Faculdade de Medicina de Ribeirão Preto Ribeirão Preto SP Brazil Faculdade de Medicina de Ribeirão Preto, Universidade de São Paulo, Ribeirão Preto, SP, Brazil; 2 Universidade Estadual de Campinas Faculdade de Ciências Médicas Campinas SP Brazil Universidade Estadual de Campinas, Faculdade de Ciências Médicas, Campinas, SP, Brazil

**Keywords:** Sexual behavior, Women, Sexually transmitted infections, Aged, Sexual practices, Female sexual function index, Surveys and questionnaires

## Abstract

**Objective:**

To assess sexual behavior, use of sexually transmitted infection prevention methods and the sexual function, among women who have sex with women.

**Methods:**

This nationwide cross-sectional study was conducted from February 2023 to April 2024 using the Respondent Driven Sampling method. Women who have sex with women (WSW) with vaginas and neovagina, aged 18–40 years were included. Data on sociodemographic, clinical history, sexual behavior, and STI prevention were collected. Sexual function was assessed using the Female Sexual Function Index (FSFI).

**Results:**

A total of 371 forms were analyzed (mean age: 28.7 ± 6.9 years). 82.0% participants reported having penile-vaginal intercourse. Nearly all reported both performing (367; 98.0%) and receiving (360; 97.0%) oral sex. 351 participants (94.6%) reported receptive and 324 (87.4%) reported insertive penetration. Insertive anal sex was reported by 94 participants (25.3%), and receptive anal sex by 87 (23.4%). Only 10 participants (2.7%) reported using barrier methods during oral sex. Based on FSFI scores, 115 participants (31.0%) were at risk for sexual dysfunction.

**Conclusion:**

Most WSW in Brazil engage in oral and vaginal sex, but the report of STI prevention remains mostly limited to the male condom, with little use of barriers in oral or digital practices. HPV and hepatitis vaccination status are high. 115 participants (31.0%) scoring below the cutoff of 26.55. No significant difference in risk of development of sexual dysfunction between age, educational level, sexual orientation and gender identity.

## Introduction

Sexual orientation refers to an individual's pattern of attraction toward others and it is classified as heterosexual, homosexual, bisexual or asexual, according to gender of those involved.^(1)^ Sexual behavior, in turn, does not always align with self-identified sexual orientation, as individuals may occasionally engage in sexual activity with people of the same gender without identifying as homosexual or bisexual. In order to inclusively address all individuals who identify as women and engage in sexual relations with other women, whether lesbian or bisexual, this study adopted the term women who have sex with women (WSW), encompassing individuals with a vulva or neovulva, vagina or neovagina.^(2)^

The sexual behaviors and practices of individuals in heterosexual relationships are already well documented in the literature; however, knowledge regarding these practices among WSW remains limited.^(3)^ Similarly, there is a lack of data on the use of contraceptive methods and the prevention of sexually transmitted infections (STI) within this population. The sharing of sexual objects, such as dildos and vibrators, during practices including anal, oral, oral-anal, and oral- vaginal sex, often occurs without the use of protection.^(4)^ This behavior may increase the risk of exposure to sexual health harms, with low adherence to preventive measures being a potential contributing factor. In a cohort study involving 1,557 WSW, low rates of barrier method use were observed during genital stimulation and during the use of sexual devices (11.3% vs. 34.4%, respectively). Additionally, more than 90% of participants reported previous sexual activity with men, often without protection. Another study found that over 80% of WSW had never used protection during oral or digital sex, which may help explain the elevated prevalence of bacterial vaginosis observed in this population.^(3-5)^

Notably, WSW faces significant barriers in accessing professional guidance on sexual health, reflecting a gap in the training of health professionals—both at the undergraduate level and during gynecology and obstetrics specialization—regarding comprehensive care for this population.^(6)^ As a result, WSW often receive less comprehensive gynecological care than heterosexual women, particularly in areas such as contraceptive counseling, the use of barrier methods during sexual activity, STI screening and access to reproductive health services.^(7)^ These disparities contribute to the increased vulnerability of this population and underscore the need for targeted educational initiatives and professional training to promote sexual health among WSW.^(8)^

Regarding the assessment of sexual function in lesbian women, a recent review identified that lesbian and bisexual women report experiencing more orgasms with their partners compared to heterosexual women.^(9)^ Additionally, perceptions of overall sexual functioning differ between heterosexual and homosexual women. However, factors such as internalized homophobia and societal prejudice may contribute to these perceptions, potentially affecting the way lesbian and bisexual women experience and report their sexual functioning.^(10)^ Studies have also evaluated the impact of sexual orientation on gynecological care, showing that these women tend to conceal their sexual orientation and often do not receive appropriate guidance regarding reproductive health.^(11)^ National data show significant variations in prevalence estimates, do not use validated instruments for data collection, and primarily focus on the difficulties faced by these women through qualitative approaches.^(11)^ In this context, this study aimed to assess sexual behavior, and use of STI prevention methods and the sexual function, among WSW, providing quantitative data at the national level.

## Methods

This nationwide descriptive cross-sectional study was conducted from February 2023 to April 2024 and involved online data collection using the Respondent Driven Sampling method. Eligible participants included WSW with vaginas, aged 18–40 years, as well as individuals who identified as women and reported having had sexual relations with other women within at least one month prior to completing the online questionnaire. Pregnant or postpartum women, and transgender women who had not undergone gender-affirming surgery (neovulvovaginoplasty), were excluded. Due to the nature of online data collection, participant eligibility could only be confirmed after data extraction from the platform. Only individuals who fully met the inclusion and exclusion criteria were considered for data analysis.

The online questionnaire was created using the Google Forms^®^ platform (https://docs.google.com). The RDS method was adopted, whereby invited participants were encouraged to share the form with acquaintances to facilitate recruitment. Form dissemination and promotion of the study were conducted via email and digital messaging, with support from LGBTQIAPN+ non-governmental organizations (NGOs). Recruitment also took place on social media platforms, including Instagram, WhatsApp, Telegram, and Facebook, using an announcement containing the following message: We are conducting a national scientific study, in collaboration with the *Faculdade de Medicina de Ribeirão Preto – Universidade de São Paulo*, on the sexual behavior of women who have sex with women. The aim is to characterize the sexual practices of this population. Individuals interested in participating were invited to contact the lead researcher via Instagram, WhatsApp, email, or Facebook for further information.

A semi-structured questionnaire was developed by the authors, comprising 16 multiple-choice questions covering demographic characteristics (age, education, race/color, and gender) and clinical aspects (number of partners, use of barrier methods, etc.). The Female Sexual Function Index (FSFI) was also applied to assess the participants’ sexual function.^(12)^ In order to prevent duplicate responses, the "Single Response" option was enabled in the form settings, restricting multiple submissions from the same cell phone number or email address. After providing informed consent, the participants were redirected to a new window to complete the semi-structured questionnaire, which began with screening questions to verify eligibility based on the inclusion criteria. Only those who gave consent could proceed to the subsequent sections. Upon completing the semi-structured questionnaire, the participants answered the FSFI questionnaire to assess the risk of sexual dysfunction. A pilot study was conducted with 10 women to evaluate their comprehension of the questions and estimate the time required to complete the 35 items (semi-structured questionnaire + FSFI). The results indicated a maximum completion time of 10 minutes.

Participants who chose to withdraw from the study, even after submitting the completed form, could request revocation by contacting the lead researcher via email, phone, or WhatsApp, as specified in the informed consent form. Consent to participate was considered granted upon submission of the completed questionnaire.

The questionnaire included the following *closed-ended variables*: age (in years: < 25; 25–38; > 38), educational level (never studied, incomplete elementary, complete elementary, incomplete high school, complete high school, incomplete higher education, complete higher education, Master's, or Doctorate); sexual orientation, self-referred as lesbian, bisexual, heterosexual, asexual, or other; sexual practices currently or previously engaged in; use of barrier method during oral, vaginal, and anal sex for STI protection; use of barrier method during intercourse involving sexual objects for STI protection; vaccination status for HPV, hepatitis B, and hepatitis A and ever had an STI. The questionnaire included the following *open-ended variables:* self-reported barrier methods against STI and reported STI.

The participants completed the FSFI questionnaire to assess their risk of sexual dysfunction. The FSFI consists of 19 questions evaluating sexual activity over the past four weeks. Each item offers six response options, where a score of 0 indicates no sexual activity, and scores ranging from 1 to 5 represent increasing levels of function or satisfaction. The questionnaire is divided into six domains: Desire (items 1 and 2), Arousal (items 3–6), Lubrication (items 7–10), Orgasm (items 11–13), Satisfaction (items 14–16), and Discomfort/Pain (items 17–19). The participants completed the FSFI questionnaire to assess their risk of sexual dysfunction. The FSFI consists of 19 questions evaluating sexual activity over the past four weeks. Each item offers six response options, where a score of 0 indicates no sexual activity, and scores ranging from 1 to 5 represent increasing levels of function or satisfaction. The questionnaire is divided into six domains: Desire (items 1 and 2), Arousal (items 3–6), Lubrication (items 7–10), Orgasm (items 11–13), Satisfaction (items 14–16), and Discomfort/Pain (items 17–19). To calculate the domain scores, the sum of the item responses for each domain is multiplied by a specific correction factor. The total FSFI score is then obtained by summing the scores from all six domains. Final scores range from 2 to 36, with higher scores indicating a better degree of sexual function. Scores ≤ 26.55 are considered indicative of a risk for sexual dysfunction.^(13)^ The FSFI used in this study was adapted to assess the sexual function of lesbian and bisexual women, with modifications to reflect sexual practices that do not involve penile penetration.^(12)^

The sample size was calculated based on a 5% margin of error and a 95% confidence level. Assuming the target population to be effectively infinite, the minimum required number of participants was estimated to be 370. This calculation was performed using the Qualtrics^®^ online sample size calculator (https://www.qualtrics.com/blog/calculating-sample-size, appropriate for a cross-sectional study design.

In order to characterize the sample, an exploratory data analysis was performed using measures of mean and standard deviation. Qualitative variables were summarized using absolute and relative frequencies. Associations involving the FSFI scores and clinical and sociodemographic variables were tested using chi-square test. All analyses were conducted using SAS version 9.4.

This project was submitted to and approved by the Research Ethics Committee of the Clinics Hospital of the Ribeirão Preto Medical School, University of São Paulo (CAAE # 65057722.5.0000.5440), and all participants provided informed consent (5.830.838). The practical activities of this study were conducted in accordance with the ethical standards outlined in Resolution No. 466/2012 of the National Health Council for research involving human beings. All data are protected under the General Data Protection Law, with access to the collected information restricted to the research team.

## Results

**Figure 1:**
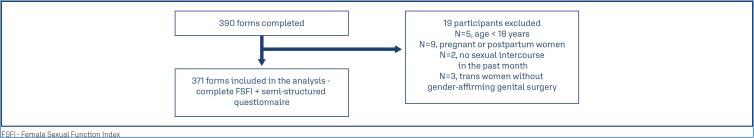
Flowchart of the included participants

A total of 390 participants completed the questionnaires between February 2023 and April 2024, of which 371 were included in the final analysis (Figure 1). The study sample consisted of 371 women from different regions of Brazil. Most participants were concentrated in the Southeast region (n=261), followed by the South (n=32), Center-West (n=41), and Northeast (n=31) regions. The North region had the lowest representation, with only 6 participants (Table 1).

**Table 1:** Sociodemographic characteristics, sexual practices, use of barrier methods, and history of sexually transmitted infections (STI) among women who have sex with women (WSW) (n=371)

Variables		n(%)
Has engaged in penile-vaginal intercourse		304(82.0)
Sexual orientation		
	Bisexual	199(53.6)
	Heterosexual	6(1.6)
	Lesbian	157(42.6)
	Other	9(2.4)
Education		
	Complete high school	32(8.6)
	Incomplete higher education	101(27.2)
	Complete higher education	197(53.0)
	Master's degree	31(8.3)
	Doctorate	10(2.7)
Sexual practices		
	Performs oral sex	367(98.0)
	Receives oral sex	360(97.0)
	Receptive vaginal penetration	351(94.6)
	Insertive vaginal penetration	324(87.4)
	Insertive anal sex	94(25.3)
	Receptive anal sex	87(23.5)
Barrier methods against STIs self-reported		
	Male condom	342(92.2)
	Dental dams	22(6.3)
	Latex gloves	15(4.3)
	Latex panties	3(0.9)
Use of barrier method		
	Oral sex	10(2.7)
	Anal sex	62(16.7)
	Tongue and finger	33(8.9)
	Sex toys	121(32.6)
HPV Vaccination Status		
	Yes	199(59)
	No	138(40.9)
Hepatitis A and B Vaccination Status		
	Yes	319(94.6)
	No	18(5.3)
Ever had an STI		
	No	298(80.3)
	Unaware	22(5.9)
	Yes	51(13.7)
Reported STI		
	Syphilis	2(0.5)
	Trichomoniasis	6(1.6)
	Chlamydia	3(0.8)
	Genital herpes	8(2.1)
	HPV	23(6.2)

STI - sexually transmitted infection; HPV - human papillomavirus

The mean age of the participants was 28.7 ± 6.9 years, and the majority were from southeastern Brazil (260, 95.0%). A total of 238 (64.2%) reported having completed higher education, and 199 (53.6%) self-identified as bisexual. The sociodemographic, clinical, and sexual behavior characteristics of the participants are detailed in table 1. The majority (82.0%) of study participants reported having engaged in penile-vaginal intercourse. Regarding sexual orientation, 199 (53.6%) identified as bisexual, 157 (42.6%) as lesbian, 6 (1.6%) as heterosexual, and 9 (2.4%) reported other orientations. Sexual practices were diverse, almost all participants reported both performing (367; 98.0%) and receiving (360; 97.0%) oral sex. The majority also reported engaging in vaginal intercourse, with 351 (94.6%) reporting receptive and 324 (87.4%) insertive vaginal penetration. Additionally, 94 (25.3%) participants reported engaging in insertive anal sex, and 87 (23.4%) reported receptive anal sex. With respect to self-reported of barrier methods for the prevention of STI, male condoms were the most widely recognized (242; 92.2%), followed by dental dams (22; 6.3%), latex gloves (22; 4.3%), and latex panties (3; 0.9%). The use of barriers for STI varied according to sexual practice. One-hundred and twenty-one (32.6%) participants reported its use in sex toys, 62 (16.7%) during anal sex, 33 (8.9%) during stimulation with tongue or fingers, and 10 (2.7%) during oral sex. Regarding vaccination status, 199 (59.0%) of participants had received the HPV vaccine, while 319 (94.6%) had been vaccinated against hepatitis. As for STI history, 298 (80.3%) reported ‘never having had an STI’. The most commonly reported infections were HPV (23; 6.2%).

The mean total FSFI score in our sample was 26.90 ± 8.2, with 115 participants (31.0%) scoring below the cutoff of 26.55. No significant difference in risk of development of sexual dysfunction between age, educational level, sexual orientation and gender identity. The sexual function characteristics of the participants, as assessed using the FSFI, are presented in table 2.

**Table 2:** Comparison of clinical and sociodemographic variables according to the risk of sexual dysfunction based on the FSFI

Variables	High risk for SD	Low risk for SD	p-value
	n(%)	n(%)	
Age (years)			0.77
< 25	37(32)	78(68)	
25-38	69(36)	162(64)	
>38	9(30)	16(70)	
Educational level			0.99
Doctorate	3(30)	7(70)	
Master	9(29)	22(71)	
Complete higher education	62(31)	135(69)	
Incomplete higher education	31(31)	70(69)	
Complete high school	10(31)	22(69)	
Sexual orientation			0.30
Bisexual	60(30)	139(70)	
Heterosexual	4(67)	2(33)	
Lesbian	48(31)	109(69)	
Other	3(33)	6(67)	
Gender identity			0.37
Agender	0(0)	2(100)	
Female	110(31)	248(69)	
Gender fluid	3(75)	1(25)	
Neutral	1(50)	1(50)	
Non-binary	1(50)	3(75)	
Other	0(0)	1(100)	

FSFI - female sexual function index; Data presented as absolute numbers (relative frequencies) of the sample; p, p-value. High risk for SD (FSFI 26,55); Low risk for SD (FSFI > 26,55)

## Discussion

This study aimed to assess sexual behavior, use of STI prevention methods and sexual function among WSW. Our study included 371 women from various regions of Brazil, with a predominance from the Southeast. The majority identified as bisexual or lesbian and reported diverse sexual practices, including high rates of oral and vaginal intercourse. While self-reported of male condoms was high, awareness and use of other STI prevention methods, such as dental dams and gloves, were notably low. Most participants had never been diagnosed with an STI, and HPV was the most frequently reported infection. Although nearly one-third of the sample scored below the FSFI cutoff suggestive of sexual dysfunction, no significant association was found between sexual dysfunction and variables such as age, education, sexual orientation, or gender identity.

Oral and vaginal sex were reported as sexual practices by nearly all participants. These results are in accordance to a study published in 2024, which evaluated 723 WSW, and identified that 98.2% of them perform oral sex and 89.1% insertive genital intercourse. The lack of visibility regarding the diversity of sexual practices among women contributes to the persistence of this unfounded notion that penetration is absent from lesbian sexuality.^(9)^ According to our results, more de 90% of WSW receives vaginal penetration. Additionally, some women who self-identify as lesbian report previous sexual activity with individuals who have penises. This misconception reinforces the idea that penetrative sex is exclusive to heterosexual relationships, overlooking the plurality of sexual experiences within the lesbian population. Such a reductionist perspective has direct implications for the sexual health of lesbian women, often leading to neglect in preventive care, such as the use of protective measures against STIs and screening for cervical cancer.^(6,9)^

Furthermore, these taboos impose barriers to open discussions about lesbian sexuality, hindering recognition of its diversity and negatively impacting both sexual education and access to appropriate healthcare for this population.^(5)^ In the present study, WSW were found to engage in anal sex. Otherwise, different from our results, the practice of anal sex was reported by 54.8% of lesbians and 64.4% of bisexuals in this Italian women.^(14)^ This difference may be related to cultural factors and stigma surrounding the practice. Some women may feel embarrassed or report pain and complications such as bleeding, while others describe the practice as enhancing intimacy and limiting it to specific partners.^(15)^ This finding highlights important nuances in sexual practices among these groups and underscores the diversity of sexual behaviors, regardless of sexual orientation.^(16-18)^

Regarding STI prevention, despite the high educational level of the study participants and widespread the use of barrier methods such as condoms, adherence to their use during penetrative sexual practices was low. Previous studies have shown that risk perception plays a crucial role in adherence to preventive measures.^(16)^ According to the NIS, to reduce the risk of STI, it is recommended to use a new condom on sex toys for each partner or when switching between different body sites, and to wash the toys with soap and water between uses. During oral sex, WSW should avoid contact if there are cuts or sores in the mouth or on the lips, or use dental dams to cover the anus or vulva. For vaginal or anal fisting, latex gloves and generous amounts of water-based lubricant are advised.^(17)^ In addition to a sense of invulnerability, the non-use of condoms is also associated with misinformation, such as the belief that oral sex does not transmit STIs, the misconception that it is unnecessary to change condoms when switching the site of penetration or partners, and the perception that condom use is mainly reserved for casual or non-regular partners.^(18)^

A review in 2022, emphasizes that although WSW are often perceived as a low-risk population, STI rates can be notably high, especially among bisexual women or those with multiple sexual partners.^(19)^ Another study, which included African American women aged 18 to 45 years, found differing STI rates by sexual orientation: 66% of bisexual women and 45% of lesbians had a history of STI, indicating a higher prevalence among bisexual participants.^(20)^ In Brazil, Pinto et al. also reported a significant infection rate among WSW, with 33.8% of participants testing positive for HPV and 7% for hepatitis B virus, highlighting the spectrum of infections affecting this population.^(21)^

In Brazil, vaccination against HPV is offered through *Sistema Único de Saúde* (SUS) and was incorporated into the National Immunization Program (PNI) in 2014. Currently, the vaccine is available to both girls and boys aged 9 to 14 years, with a single-dose schedule recommended for this age group, and individuals who are survivors of sexual violence, immunosuppressed, PrEP users, or have cervical pre-neoplastic lesions, in a three-dose schedule.^(22)^ The fact that nearly 60% of the study participants were vaccinated against HPV may be related to factors such as the high educational level of the sample, which is generally associated with greater awareness of the importance of vaccination and better access to health services. Additionally, vaccination campaigns carried out in schools and primary healthcare units may have contributed to this coverage.^(23)^ The high vaccination rate against hepatitis (94.6%) highlights the importance of immunization as a preventive strategy. Oral-anal sex (anilingus), reported by 20% of our sample, is a recognized risk factor for hepatitis A. Therefore, vaccination against hepatitis A is particularly relevant for individuals who engage in this practice, as it significantly reduces the risk of infection.^(4)^

In our study, 31.0% of women who have sex with women had a total FSFI score below the cutoff of 26.55, indicating a risk for sexual dysfunction, a finding consistent with the wide prevalence range reported in the literature (13.3% to 79.3%).^(24)^ This concordance underscores the importance of assessing sexual function in WSW, a population often underrepresented in research. Moreover, it highlights the need for specific studies exploring the affected domains and their particularities. In our study, age was not associated with the risk for sexual dysfunction. This finding contrasts with what would be expected, as sexual dysfunction is known to correlate with age in cisgender women over the life course.^(24)^ One potential source of bias in this analysis is the composition of our sample, which consisted predominantly of young women (mean age 28.7 ± 6.9 years) and was not stratified by age. This may have resulted in a recruitment bias.

Addressing sexual function in this population is challenging due to a combination of cultural stigmas, psychological barriers, gender inequality, prejudice, and a lack of preparedness within healthcare services. Taboos and myths surrounding sexuality often generate feelings of guilt and fear of judgment, making open discussions and help-seeking more difficult. Additionally, inadequate training of health professionals and limited consultation time compromise the quality of care.^(12-14)^ Factors such as religious influence, gender inequality, and stigma toward sexual diversity and the sexuality of older individuals further hinder inclusive approaches. Regarding sociodemographic characteristics, no association was found between sexual dysfunction and educational level, as most participants had at least eight years of schooling. Previous studies suggest that higher education may be associated with lower rates of sexual dysfunction.^(25)^

The results of this study highlight behaviors and vulnerabilities that cannot be understood solely from a biological perspective but must be analyzed within social contexts marked by homophobia, invisibility, and misinformation. The low adherence to STI prevention methods, even among highly educated women, suggests not only gaps in access to information but also symbolic and cultural barriers that shape the construction of sexuality among women who have sex with women. Institutional homophobia excludes these women from health indicators, monitoring systems, and public health programs, which directly contributes to misinformation and lack of access. The American College of Obstetricians and Gynecologists recommends adapting gynecologic care to better support LGBTQIA+ women by using inclusive language, updating intake forms to reflect sexual orientation and behavior, clearly posting nondiscrimination policies in the office, and providing educational materials and community resources for patients and their families.^(26)^

One limitation of the present study was the demographic distribution of the evaluated sample, with the vast majority of participants concentrated in the Southeast region of Brazil. In contrast, a study published in 2018 reported a higher concentration of participants from the Midwest region (28.4%) compared to other areas.^(11)^ This discrepancy may be attributed to differences in recruitment strategies. In addition, it is imperative that future studies use recruitment methods and more representative samples, considering regional and socioeconomic diversity, to generate data that can support effective public policies for the promotion of sexual health in this population. Another notable bias relates to the relatively high educational level of our participants. Women with higher educational attainment generally have better access to information about sexual health, which may help explain the higher self-reported STI prevention methods in our sample.

## Conclusion

Most WSW in Brazil engage in oral and vaginal sex; however, the report about barrier methods for STI prevention were largely limited to the male condom, and during oral sex or digital stimulation, barrier methods are generally not used. Vaccination status for HPV and hepatitis are relatively high. No significant difference in risk of development of sexual dysfunction between age, educational level, sexual orientation and gender identity. One possible source of bias in this analysis is the composition of our sample, which was predominantly made up of young women and was not stratified by age. These results highlight the need for tailored sexual health education and clinical care that recognize diverse sexual practices and improve access to prevention. Increasing visibility of WSW's sexual health can enhance gynecological care and reduce disparities.

## Data Availability

The research data are described in the article presented.
